# Global, regional, and national prevalence, disability adjusted life years, and time trends for refraction disorders, 1990–2019: findings from the global burden of disease study 2019

**DOI:** 10.1186/s12889-021-11648-1

**Published:** 2021-09-06

**Authors:** He-Yan Li, Yue-Ming Liu, Li Dong, Rui-Heng Zhang, Wen-Da Zhou, Hao-Tian Wu, Yi-Fan Li, Ya-Xing Wang, Wen-Bin Wei

**Affiliations:** 1 Beijing Tongren Eye Center, Beijing Key Laboratory of Intraocular Tumor Diagnosis and Treatment, Beijing Ophthalmology & Visual Sciences Key Lab, Medical Artificial Intelligence Research and Verification Key Laboratory of the Ministry of Industry and Information Technology, Beijing Tongren Hospital, Capital Medical University, 1 Dong Jiao Min Lane, Beijing, 100730 China; 2 Beijing Institute of Ophthalmology and Beijing Ophthalmology and Visual Science Key Lab, Beijing Tongren Eye Center, Beijing Tongren Hospital, Capital Medical University, Beijing, China

**Keywords:** Refraction disorders, Health burden, Vision loss, Disability adjusted life year

## Abstract

**Background:**

To evaluate global burden of refraction disorders by year, age, region, gender, socioeconomic status and other national characteristics in terms of disability adjusted life years (DALYs) and prevalence from Global Burden of Disease (GBD) study 2019 and World Bank Open Data 2019.

**Methods:**

Global, regional, and national DALY numbers, crude DALY rates, age-standardized DALY and prevalence rates of refraction disorders were acquired from the GBD study 2019. Mobile cellular subscriptions, urban population, GDP per capita, access to electricity and total fertility rate were obtained from the World Bank to explore the factors that influenced the health burden of refraction disorders. Kruskal-Wallis test, linear regression and multiple linear regression were performed to evaluate the associations between the health burden with socioeconomic levels and other national characteristics. Wilcoxon Signed-Rank Test was used to investigate the gender disparity.

**Results:**

Globally, age-standardized DALY rates of refraction disorders decreased from 88.9 (95% UI: 60.5–120.3) in 1990 to 81.5 (95% UI: 55.0–114.8) in 2019, and might fall to 73.16 (95% UI: 67.81–78.51) by 2050. Age-standardized prevalence rates would also reduce to 1830 (95% UI: 1700–1960) by 2050, from 2080 (95% UI: 1870–2310) in 1990 to 1960 (95% UI: 1750–2180) in 2019. In low SDI region, age-standardized DALY rates (equation: *Y* = 114.05*X + 27.88) and prevalence rates (equation: Y = 3171.1*X + 403.2) were positively correlated with SDI in linear regression respectively. East Asia had the highest blindness rate caused by refraction disorders in terms of age-standardized DALY rates (11.20, 95% UI: 7.38–16.36). Gender inequality was found among different age groups and SDI regions.

**Conclusion:**

Health burden of refraction disorders decreased in recent years, and may continue to alleviate in the next three decades. Older ages, females and lower socioeconomic status were associated with higher refraction disorders health burden.

## Synopsis

We explored the health burden caused by refraction disorders using the GBD data 2019, which requires more public health policies and studies.

## Background

Refraction disorders affect a large proportion of the world population, and is the major cause of visual impairment and second cause of blindness [1]. In 2020, 1.1 billion people get distance visual impairment or uncorrected presbyopia worldwide, of whom 596 million has distance visual impairment with 43 million are blind [2, 3]. By 2050, an estimated 1.8 billion people will suffer from vision loss due to refraction disorders. Refraction disorders may reduce educational opportunities, productivity, and overall quality of life. The productivity loss has been estimated at $202 billion per annum after adjustment for country specific labor force participation and employment rates, which are mainly caused by vision loss [4, 5].

Visual impairment due to refraction disorders is a preventable cause of disability. The health burden of refraction disorders has been assessed by disability-adjusted life years (DALYs). Encouragingly, more than 90% people with visual impairment caused by refraction error can be prevented with existing cost-effective interventions [6]. The year of 2020 marks the culmination of a global initiative to eliminate avoidable blindness, VISION 2020: The Right to Sight, preceded by the publication of a WHO World Report on Vision [7] and the 73rd World Health Assembly resolution on integrated people-centered eye care.

Refraction disorders remain a major medical challenge around the world. Recent studies have revealed that refraction disorders, especially myopia have become a global pandemic. In East Asia, about 80% of 20-year-olds now have myopia, compared with 20–30% in the mid-twentieth century [8]; cases of severe myopia—with complications such as myopic macular degeneration, retinal detachment, and glaucoma—are presenting at much younger ages [8]. This study evaluated the health burden of refraction disorders in terms of prevalence and DALYs by using the most recent data from the GBD 2019 study, and we tried to find the factors that influenced the burden globally by using more data from World Band Open Data 2019. We aimed to raise the attention of the public to prevent refraction disorders at younger age and provide more qualitive eye care services. If this decade is truly to be the Decade of Action for Sustainable Development Goals (SDGs), equity and quality of eye care services must be at the heart of all we do to achieve the ambition.

## Methods

### Data extraction

The most common refraction disorders included myopia, hyperopia, and astigmatism. Methods to generate DALYs estimates in the GBD 2019 study have been previously described [9]. Generally, DALYs were the sums of years lived with disability (YLDs) and years of life lost (YLLs) because of premature death, while DALYs estimates for refraction disorders were equal to YLDs, according to the GBD 2019 study [9]. The following data regarding refraction disorders were acquired from the Global Health Data Exchange (http://ghdx.healthdata.org/gbd-results-tool), (1) global total and age- and gender-specific of prevalence and DALYs data, as absolute number and age-standardized rates (per 100,000 population) from 1990 to 2019, (2) global total data of DALY, as crude rates (per 100,000 population) from 1990 to 2019, (3) GBD super region’s total and gender-specific DALY data in 1990 and 2019, as age-standardized rates; (4) GBD super region’s DALY data, as age-standardized rates of causes attribute to vision loss in 1990 and 2019; and (5) Socio-demographic Index (SDI) of GBD countries in 2019. The following data were extracted from Word Bank open data (http://data.worldbank.org/) in 2019, (1) mobile cellular subscriptions (per 100 people); (2) urban population (% of total population); (3) GDP per capita (current US$); (4) access to electricity (% of population); (5) fertility rate, total (births per woman). Ethics approval and informed consent were not required for this study because of public accessibility to the data.

### Variables

The Socio-demographic Index (SDI) of GBD 2019 indicated the overall development, which included total fertility rate, lagged distributed income and education level [10]. The SDI ranged from 0 to 1, while higher SDI implicated better socioeconomic development: high SDI (> 0.81), high-middle SDI (0.70–0.81), middle SDI (0.61–0.69), low-middle SDI (0.46–0.60) and low SDI (< 0.46). Moderate and serve visual acuity (MSVI) indicated visual acuity (VA) < 6/18 but ≧ 3/60 based on Snellen chart, while the blindness was VA < 3/60 or visual field around central fixation < 10%. All vision loss equaled to the sum of different stages of vision loss [3].

### Forecasting refraction disorders burden beyond 2019

Auto-Regressive Integrated Moving Average (ARIMA) model was widely used in epidemiological study to predict future outcomes [11]. It was performed on R Statistical Software (version 4.0.3) with forecast (version 8.13) and tseries (version 0.10–48) packages. We forecasted health burden caused by refraction disorders in terms of age-standardized rates of DALY and prevalence from 2020 to 2050. In ARIMA (*p*, *d*, *q*) model, *p* represented the number of lag observations; *d* represented the number of times input raw data are different to make the model stationary; and *q* represented the size of moving average window applied to lagged observations. We established an ARIMA model to make the prediction and then tested the model.

### Statistical analyses

Age-standardized rates of DALY and prevalence were expressed as the number per 100,000 population with 95% uncertainty intervals (UIs). Wilcoxon Signed-Rank Test [12] was used to make the comparisons of gender difference in national DALY numbers and crude DALY rates for each age group. The difference of age-standardized DALY rates among five SDI regions were explored by Kruskal-Wallis test [13], followed by evaluation for multiple comparisons between genders using Wilcoxon Signed-Rank Test with Bonferroni Correction. Linear regression analyses were used to investigate the effects of national SDI on age-standardized rates of DALY and prevalence. Multiple linear regression analysis was performed to investigate the influence of five other variations acquired from Word Bank on age-standardized rates of DALY and prevalence. All statistical analyses were performed using R Statistical Software (version 4.0.3; R Foundation for Statistical Computing, Vienna, Austria). *P* value less than 0.05 was considered statistically significant.

## Results

### Global trends in DALY of refraction disorders

**Fig. 1:**
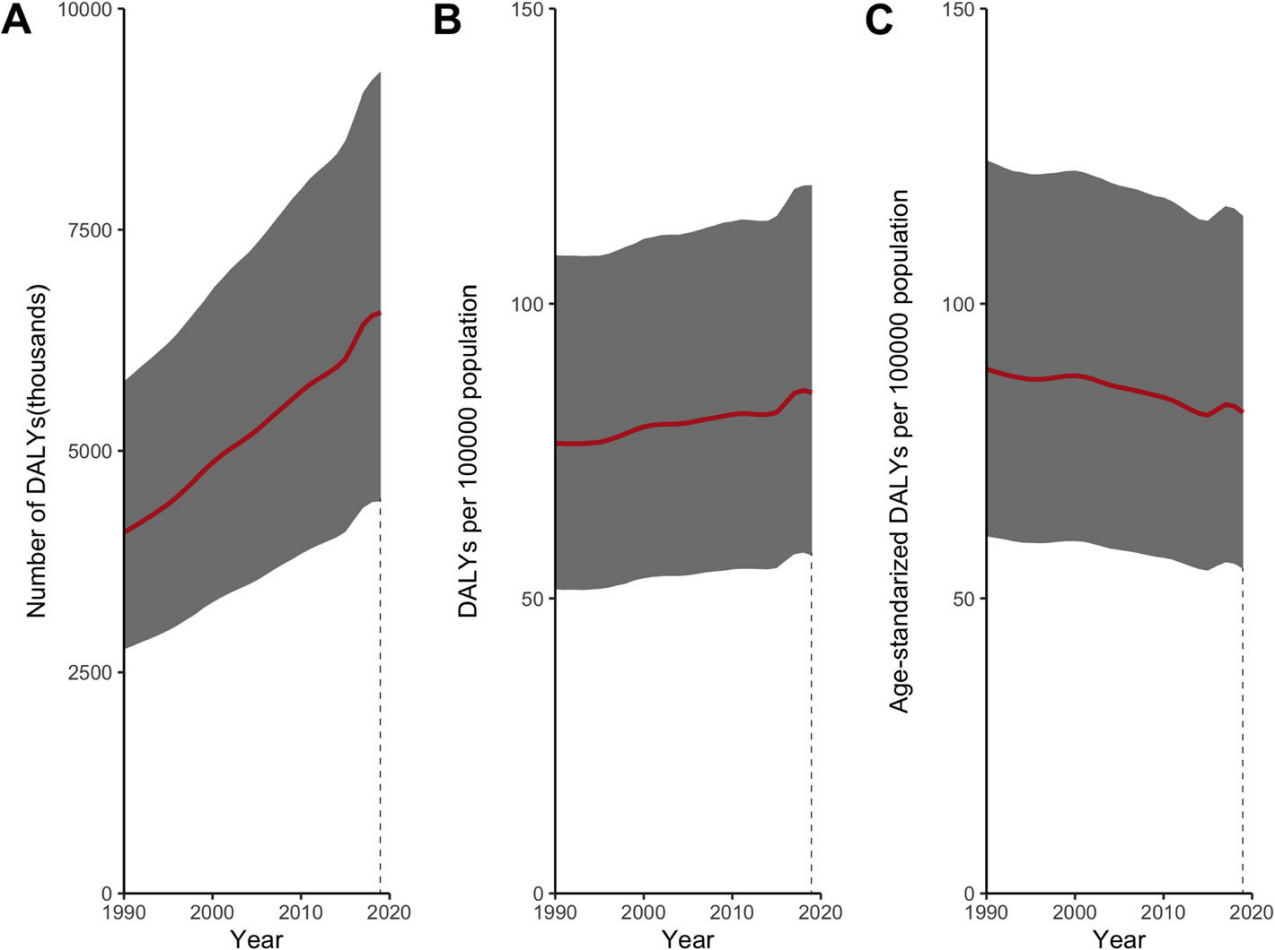
Trends in global burden of refraction disorders in terms of DALY numbers (A), crude DALY rates (B), and age-standardized DALY rates (C), from 1990 to 2019. *Sbade* areas represent 95% uncertainty intervals. DALYs = disability adjusted life years

GBD included 204 countries in DALY numbers, crude and age-standardized DALY rates in 2019. The global DALYs of refraction disorders increased by 61.0% from 4.1 (95% UI: 2.8–5.8) million in 1990 to 6.6 (95% UI: 4.4–9.3) million in 2019 (Fig. 1A). After accounting for population growth, the crude DALY rates rose slightly from 76.3 (95% UI: 51.5–108.2) in 1990 to 84.9 (95% UI: 57.3–120.1) in 2019 (Fig. 1B). In terms of age-standardized DALY rates, it fell by 8.3% from 88.9 (95% UI: 60.5–120.3) in 1990 to 81.5 (95% UI: 55.0–114.8) in 2019 (Fig. 1C).

### Refraction disorders burden stratified by age and gender

**Fig. 2:**
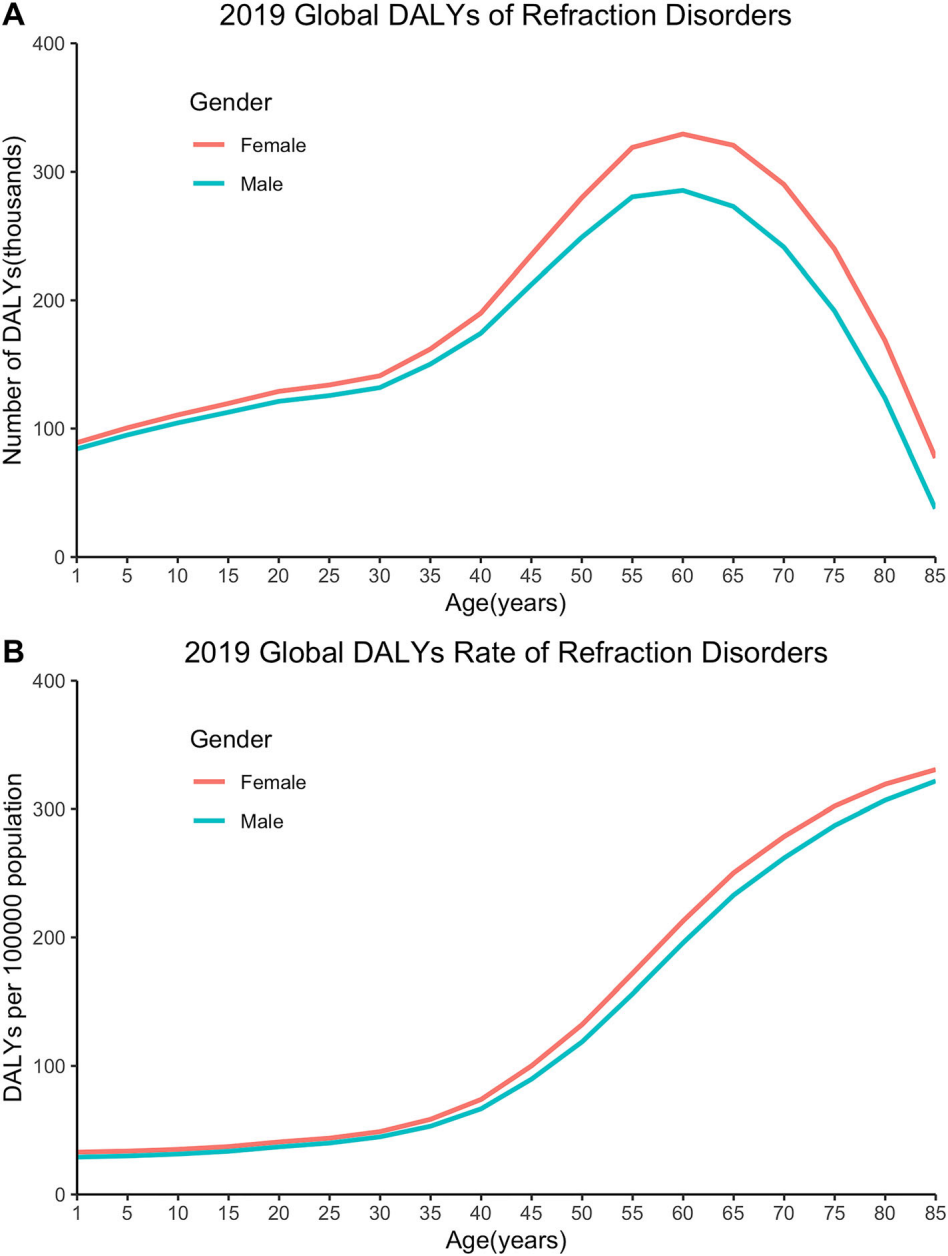
Global burden of refraction disorders in terms of DALY numbers (A), crude DALY rates (B) by age and gender in 2019. DALYs = disability adjusted life years

DALY numbers, crude and age-standardized DALY rates stratified by age and gender were available for 204 countries in 2019. Wilcoxon Signed-Rank Test showed significant gender difference in global DALY numbers and crude DALY rates in different age groups (*p* < 0.05). The gender inequality of DALYs was small between 1 to 30 years old, while peaked at in the 65–70 age group, with DALYs of 0.35 (95% UI: 0.23–0.50) million among women versus 0.30 (95% UI: 0.20–0.43) million among men (Fig. 2A). Global DALY crude rates were also higher in older females than in males of the same age, and the biggest difference was observed in the 60–65 age group, with crude DALY rates of 214.76 (95% UI: 138.07–314.05) among women versus 196.63 (95% UI: 127.87–287.89) among men (Fig. 2B).

### Predicted global burden of refraction disorders

**Fig. 3:**
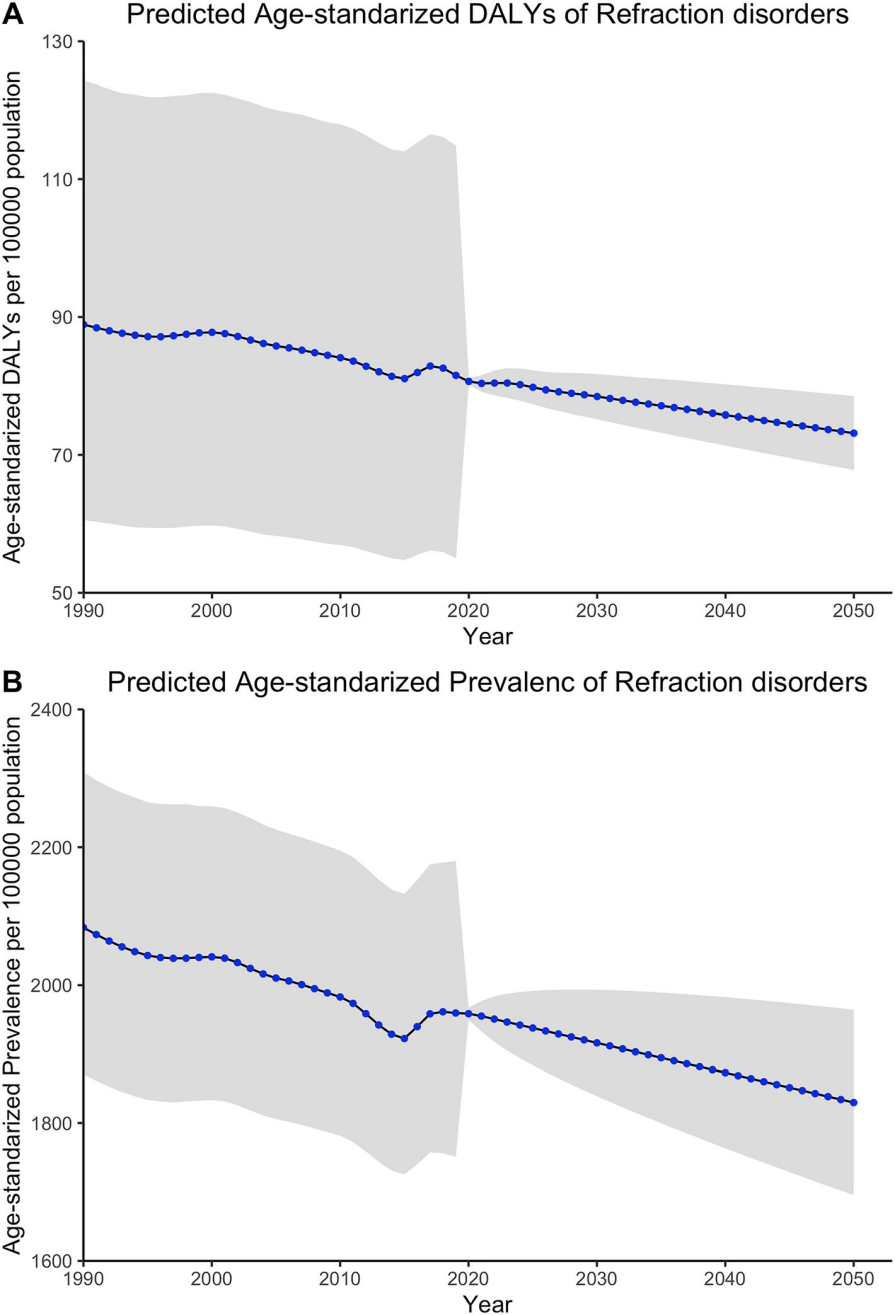
Global burden of refraction disorders from 1990 to 2050 in terms of age-standardized DALY rates (A), and age-standardized prevalence rates (B). *Sbade* areas represent 95% uncertainty intervals. DALYs = disability adjusted life years

ARIMA model was used to predict the global burden of refraction disorders in terms of age-standardized rates of DALY and prevalence beyond 2019. Generally, both age-standardized DALY rates and prevalence rates declined from 1990 to 2019, but there was an increase in recent years since 2015. Decreased burden was expected towards 2050 by ARIMA (2,1,1) model in terms of age-standardized DALY rates to 73.16 (95% UI: 67.81–78.51). While another ARIMA (0,1,2) model revealed that age-standardized prevalence rates would also reduce to 1830 (95% UI: 1700–1960) (Fig. 3).

### Refraction disorders burden by socioeconomic status

**Fig. 4:**
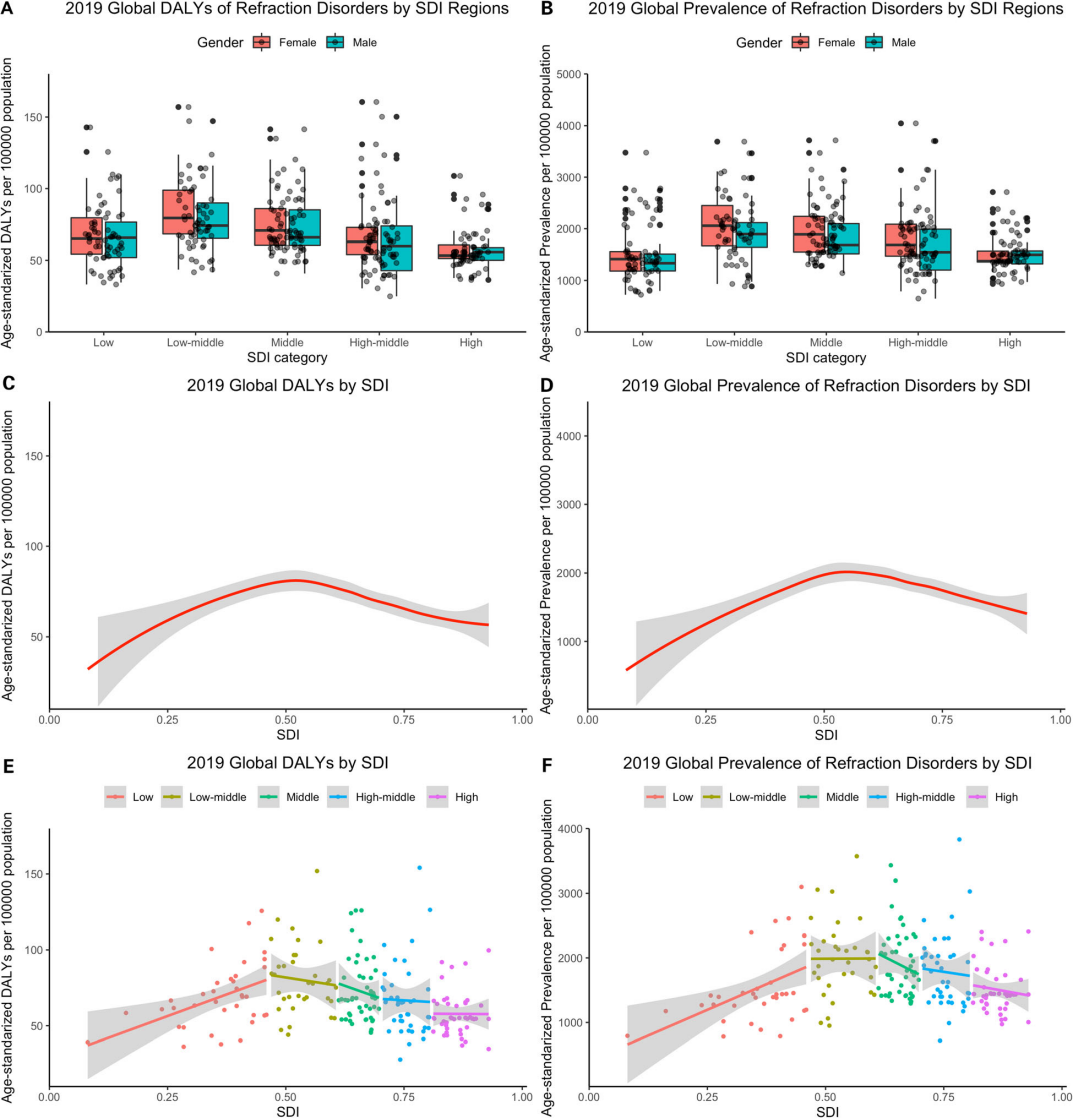
Health burden of refraction disorders in SDI regions in 2019. Gender-specific burden in terms of age-standardized DALY rates (A), and age-standardized prevalence rates (D) in 174 countries. Age-standardized DALY rates (B), and age-standardized prevalence rates (E) by SDI. Age-standardized DALY rates (C), and age-standardized prevalence rates (F) in different SDI regions. *Sbade* areas represent 95% uncertainty intervals. SDI = socio-demographic index; DALYs = disability adjusted life years. ****p < 0.0001, ns: no significant (with Bonferroni Correction)

Data of SDI were available for 179 countries in 2019 and were classified into five groups, including high (*n* = 34), high-middle (*n* = 41), middle (*n* = 40), low-middle (*n* = 31), low (*n* = 33) SDI countries and territories. Kruskal-Wallis tests revealed that age-standardized DALY rates significantly different among countries with different SDI regions in 2019 (χ^2^_(4)_ = 57.86, *p* < 0.001). At the same time, there was strong difference of age-standardized prevalence rates among countries in different SDI regions in 2019 (χ^2^_(4)_ = 56.50, *p* < 0.001). Wilcoxon Signed-Rank Test with Bonferroni Correction showed gender inequality in all SDI regions in terms of age-standardized DALY rates in 2019, except in low SDI region (*p* < 0.05). We also found gender inequality in low-middle, middle, and high-middle SDI regions in terms of age-standardized prevalence rates in 2019 (*p* < 0.05). Generally, low-middle SDI region had higher both age-standardized rates of DALY and prevalence in 2019 (Fig. 4A, B). The inverted U curve depicted the association between SDI and the burden of refraction disorders in terms of age-standardized rates of DALY and prevalence, which peaked when SDI was approximately 0.5. (Fig. 4C, D) In low SDI region, age-standardized DALY rates and age-standardized prevalence rates were positively correlated with SDI in Pearson correlation (*r* = 0.462, *p* = 0.005) and (*r* = 0.474, *p* = 0.003), with linear regression (equation: *Y* = 114.05*X + 27.88) and (equation: *Y* = 3171.1*X + 403.2), respectively. While in middle SDI region, both rates decreased more rapidly with socioeconomic development than in any other SDI regions (Fig. 4E, F).

### Refraction disorders vision loss burden by GBD super regions

**Fig. 5:**
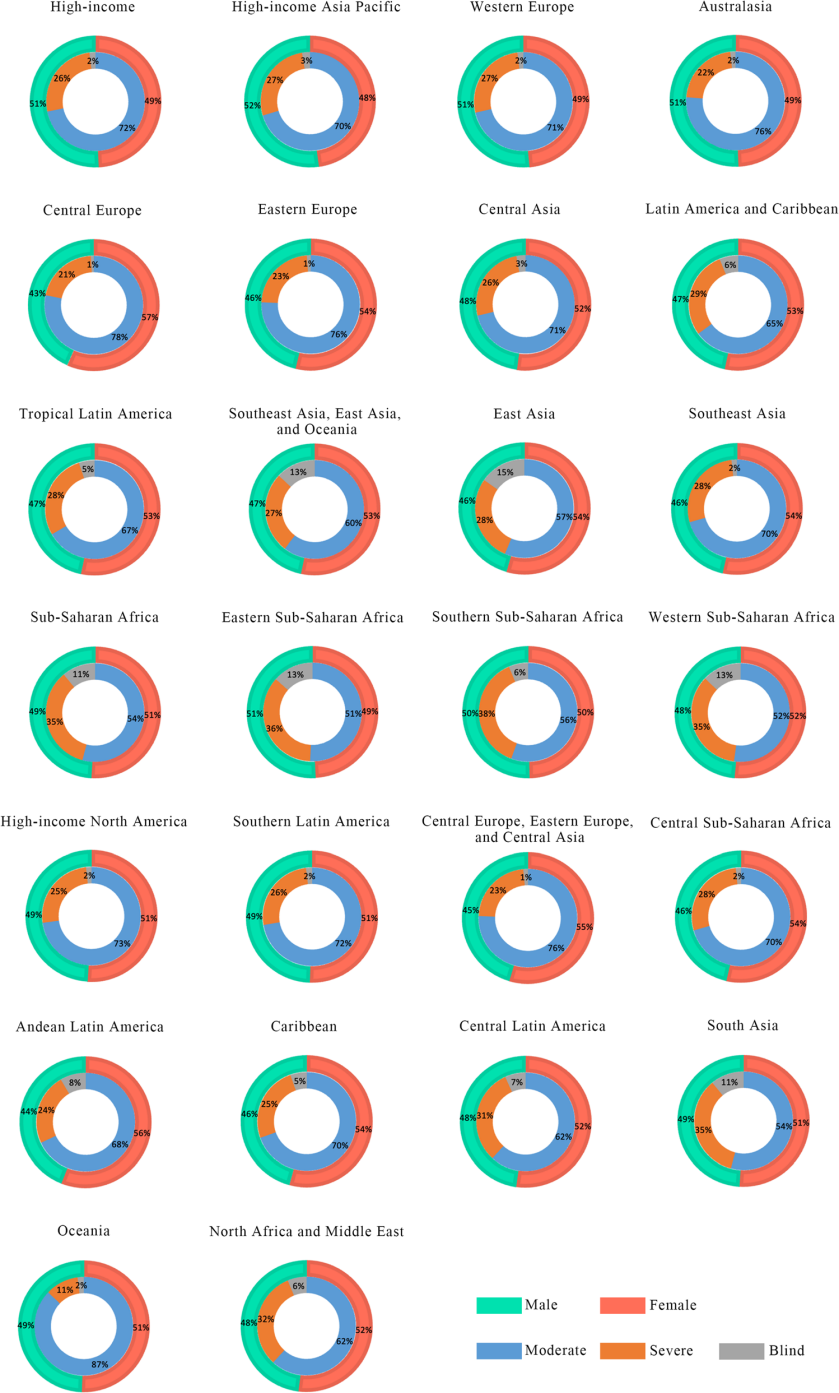
Age-standardized DALY rates of blindness, moderate and severe visual impairment associated with refraction disorders by GBD super regions and gender in 2019

The dual-ring chart depicted the proportion of gender and visual impairment burden caused by refraction disorders in 26 GBD super regions in 2019 counted by age-standardized DALY rates (Fig. 5). Generally, moderate vision loss took the majority parts in all of the GBD super regions, with Southeast Asia [50.61 (95% UI: 30.96–80.20)] and Central Europe [38.64 (95% UI: 23.61–61.25)] in the leading place in terms of age-standardized DALY rates. While East Asia [11.20 (95% UI: 7.38–16.36)] had the highest blindness rate caused by refraction disorders in terms of age-standardized DALY rates. In 22 of the 26 GBD super regions, compared to males, females suffered a slightly higher age-standardized DALY rates due to refraction disorders vision loss in 2019. Details of vision loss burden in terms of age-standardized DALY rates due to refraction disorders were showed in Table 1.

**Table 1:** Age-standardized DALY rates (per 100,000 population) of vision loss due to refraction disorders by GBD Super Region, 1990 and 2019, the Global Burden of Disease Study

GBD Super Region	1990												2019											
	All vision loss			Moderate vision loss			Serve vision loss			Blindness			All vision loss			Moderate vision loss			Serve vision loss			Blindness		
	Female	Male	Both	Female	Male	Both	Female	Male	Both	Female	Male	Both	Female	Male	Both	Female	Male	Both	Female	Male	Both	Female	Male	Both
**High-income**	56.14	57.52	57.2	38.29	41.87	40.26	16.36	14.36	15.55	1.49	1.29	1.39	54.14	55.76	55.14	37.7	41.26	39.58	15.27	13.46	14.45	1.17	1.04	1.1
High-income Asia Pacific	47.35	51.65	49.59	32.48	35.98	34.29	13.06	14.17	13.65	1.81	1.49	1.65	46.7	50.9	48.9	32.54	36.13	34.41	12.73	13.62	13.2	1.42	1.15	1.28
Western Europe	66.79	70.26	69.07	44.81	51.53	48.46	20.15	17.21	18.95	1.83	1.51	1.67	64.39	68.26	66.62	43.9	50.84	47.54	19.06	16.23	17.77	1.42	1.19	1.3
Australasia	51.99	53.86	53.06	38.4	41.5	40	12.43	11.35	11.98	1.17	1.01	1.09	52.35	53.68	53.06	38.98	41.93	40.45	12.4	10.91	11.7	0.97	0.83	0.9
High-income North America	42.5	40.65	41.82	29.71	30.21	30.05	12.02	9.67	11	0.77	0.76	0.77	42.12	40.52	41.44	29.95	30.31	30.18	11.44	9.44	10.52	0.72	0.77	0.74
Southern Latin America	77.37	75.6	76.85	53.64	54.01	54.05	21.8	19.72	20.9	1.93	1.87	1.89	72.19	70.63	71.72	51.46	51.87	51.87	19.28	17.32	18.42	1.45	1.44	1.44
**Central Europe, Eastern Europe, and Central Asia**	**79.75**	**65.74**	**74.03**	**59.02**	**50.35**	**55.34**	**19.65**	**14.11**	**17.52**	**1.09**	**1.28**	**1.18**	**76.88**	**63.94**	**71.24**	**57.62**	**49.33**	**53.91**	**18.21**	**13.49**	**16.25**	**1.04**	**1.12**	**1.08**
Central Europe	56.7	43.15	50.7	44.42	33.94	39.74	11.71	8.75	10.44	0.57	0.46	0.52	55.28	42.35	49.4	43.2	33.25	38.64	11.56	8.66	10.28	0.52	0.44	0.49
Eastern Europe	91.73	78.71	86.47	67.12	60.91	64.3	23.61	16.18	20.89	1	1.63	1.28	89.1	76.67	83.7	66.33	60.01	63.36	21.92	15.33	19.27	0.85	1.33	1.07
Central Asia	76.97	71.19	74.29	55.12	48.49	52.18	19.23	20.78	19.79	2.62	1.91	2.32	72.54	66.6	69.75	52.55	46.35	49.72	17.6	18.54	17.94	2.4	1.71	2.09
**Latin America and Caribbean**	**122.82**	**107.63**	**115.38**	**77.46**	**66.34**	**71.99**	**35.79**	**32.77**	**34.31**	**9.56**	**8.53**	**9.07**	**111.81**	**98.08**	**105.01**	**73.27**	**63.17**	**68.24**	**31.67**	**28.9**	**30.3**	**6.87**	**6.01**	**6.47**
Andean Latin America	133.71	102.49	118.42	83.42	69.09	76.36	32.96	21.86	27.55	17.33	11.53	14.51	122.57	96.29	109.62	81	68.28	74.64	30.79	20.37	25.72	10.78	7.64	9.25
Caribbean	80.7	67.99	74.44	55.44	45.18	50.38	20.52	18.01	19.3	4.74	4.8	4.77	74.13	62.49	68.37	52.02	43.06	47.56	18.36	15.8	17.11	3.75	3.64	3.69
Central Latin America	117.66	106.41	112.15	71.07	59.78	65.56	35.36	37.91	36.57	11.22	8.72	10.03	103.28	94.12	98.66	65.98	55.76	60.94	30.07	32.28	31.03	7.22	6.08	6.7
Tropical Latin America	137.6	121.61	129.72	88.72	78.14	83.46	41.27	34.79	38.16	7.61	8.68	8.11	127.2	111.26	119.31	84.41	74.57	79.45	36.55	30.65	33.71	6.24	6.04	6.15
**Southeast Asia, East Asia, and Oceania**	84.66	74.82	79.97	47.51	42.58	45.09	23.92	20.32	22.24	13.23	11.93	12.65	80.17	70.11	75.27	48.38	42.48	45.44	21.63	18.24	20.03	10.15	9.39	9.81
East Asia	83.27	70.8	77.36	43.59	37.89	40.82	25.3	19.86	22.76	14.37	13.05	13.77	79.86	66.81	73.49	45.15	37.84	41.5	23.24	18.12	20.79	11.47	10.84	11.2
Southeast Asia	84	85.67	84.61	55.6	55.36	55.35	18.76	22	20.22	9.64	8.31	9.04	72.76	73.42	72.88	51.02	50.45	50.61	15.15	17.5	16.2	6.59	5.47	6.08
Oceania	84.24	83.91	84.05	72.98	72.73	72.84	8.93	9.03	8.97	2.33	2.15	2.24	86.15	84.09	85.07	74.77	73.4	74.05	9.53	9.05	9.28	1.84	1.65	1.74
**North Africa and Middle East**	**121.92**	**112.63**	**117.17**	**69.29**	**64.64**	**66.91**	**42.69**	**39.08**	**40.85**	**9.93**	**8.91**	**9.41**	**106.4**	**98.07**	**102.1**	**65.15**	**61.21**	**63.11**	**34.4**	**30.91**	**32.6**	**6.85**	**5.95**	**6.39**
**South Asia**	**233.65**	**202.19**	**217.17**	**126.18**	**112.68**	**119.06**	**69.26**	**57.81**	**63.28**	**38.21**	**31.71**	**34.83**	**164.42**	**150.29**	**157.24**	**97.92**	**91.4**	**94.5**	**48.02**	**41.45**	**44.77**	**18.48**	**17.44**	**17.97**
**Sub-Saharan Africa**	**64.57**	**62.47**	**63.48**	**33.53**	**32.54**	**33**	**23.02**	**22.1**	**22.54**	**8.03**	**7.83**	**7.93**	**64.06**	**61.52**	**62.82**	**34.78**	**33.24**	**34.03**	**22.47**	**21.28**	**21.9**	**6.81**	**7**	**6.89**
Eastern Sub-Saharan Africa	61.27	63.01	62.09	29.71	29.58	29.63	22.53	23.86	23.16	9.03	9.58	9.3	54.31	55.87	55	28.12	27.81	27.94	19.15	20.3	19.68	7.04	7.76	7.39
Southern Sub-Saharan Africa	75.33	75.62	75.3	40.25	42.59	41.2	29.82	28.43	29.14	5.26	4.6	4.96	69.26	69.99	69.34	37.49	40.17	38.51	27.31	25.47	26.43	4.45	4.36	4.4
Western Sub-Saharan Africa	63.18	59.59	61.3	30.99	30.69	30.79	22.38	20.07	21.2	9.81	8.83	9.31	69.89	65.78	67.92	35.95	34.88	35.43	25	22.41	23.76	8.94	8.49	8.73
Central Sub-Saharan Africa	67.1	59.58	63.58	47.71	40.8	44.49	18.02	17.58	17.81	1.37	1.2	1.29	66.69	57.76	62.57	47.65	39.95	44.15	17.87	16.78	17.31	1.17	1.02	1.1

All vision loss equaled to the sum of different stages of vision loss. *DALYs* disability adjusted life years, *GBD* Global Burden of Disease

### Refraction disorders burden by national characteristics

**Fig. 6:**
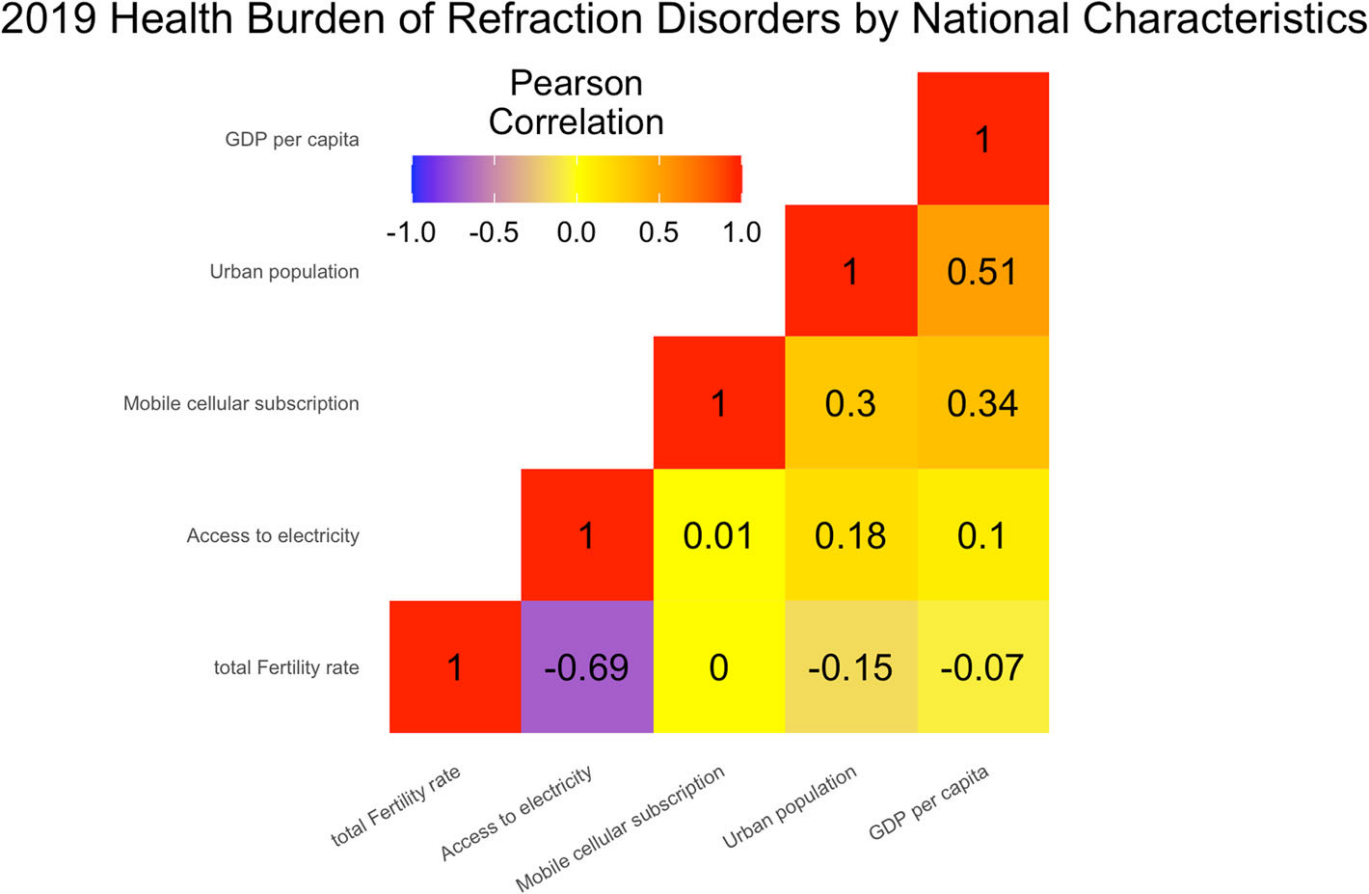
Health burden of refraction disorders by national characteristics regions in 2019 in terms of age-standardized DALY rates and age-standardized prevalence rates. Colors indicate Pearson Product-Moment Correlation Coefficient (PPMCC), with red representing Pearson’s *r* = 1, and purple representing Pearson’s *r* = −1

While multiple factors may influence the development of refraction disorders. Multiple regression analyses were used to explore the relationship between age-standardized rates of DALY and prevalence with five other factors, including mobile cellular subscriptions (β_1_), urban population (β_2_), GDP per capita (β_3_), access to electricity (β_4_) and total fertility rate (β_5_) in 174 GBD countries in 2019. The five factors explained 9.24% of the variation across countries in terms of age-standardized DALY rates (*p* < 0.05), with β_3_ = − 3.247 (*p* < 0.05). While they explained 4.03% of the variation across countries in terms of age-standardized prevalence rates (*p* < 0.05), with β_4_ = 4.932 (*p* < 0.05) (Fig. 6).

## Discussion

This study reveals the global health burden of refraction disorders by year, age, gender, region, socioeconomic status, and other national characteristics, including mobile cellular subscriptions, urban population, GDP per capita, access to electricity and total fertility rate. The DALY numbers increase, and the crude DALY rates remain stable, while the age-standardized DALY rates decline from 1990 to 2019 globally. This indicates that it is the increasing and aging population that keeps DALYs rising and the crude DALY rates stable. According to ARIMA model, the age-standardized rates of DALY and prevalence will decrease in the next three decades towards 2050. Global health burden of refraction disorders increases with age, females, and lower socioeconomic levels.

Ye’s study has found higher burden of refraction disorders in countries with lower socioeconomic status, which has been also confirmed in our study [14]. We further reveal that the health burden of refraction disorders increases before it decreases, which peaks when SDI is about 0.50 in 2019. One reason may be that people in developed countries have easier accessibility to eye care services. The average number of eye doctors per million population varies with economic development, from 3.7 per million in low income countries to 76.2 per million in high income countries [15]. Considering the quality of care, socioeconomic level is not the only factor that influence the health burden, because refraction disorders in developed countries could also be undetected [16, 17]. It is suggested that the health burden may also be affected by races, cultures, and accessibility to eye care services.

Xu’s study has revealed that China’s vision loss burden had increased more rapidly than other G20 countries from 1990 to 2019 [18]. The main cause of moderate and severe visual impairment are uncorrected refraction disorders, cataract, and macular degeneration in 2019, which were the same in 1990. Refraction disorders is the main cause of moderate visual impairment in people younger than 70 years [18]. Consist with these findings, refraction disorders are less affected by population aging than other eye diseases and the population affected are becoming younger. We also collect more data from Word Bank to further explore why health burden of refraction disorders is so heavy and happens at younger ages, and we reveal the gender disparity from 1990 to 2019.

WHO have showed that at least 2.2 billion people around the world are affected by blindness and visual impairment. 1 billion blind people caused by refraction disorders worldwide are preventable [19], which can be solved through corrective glasses or refractive surgery [20]. Uncorrected refractive disorders, cataract, age-related macular degeneration, glaucoma, and diabetic retinopathy are the leading causes of visual impairment worldwide [19]. Myopia is one of the most common eye diseases globally, with a prevalence of 10–30% in the adult population in many countries and 80–90% in young adults in parts of East and Southeast Asia [20–22]. It should be noted that myopia is the biggest burden of refraction disorders [23]. Excessive eye use, improper reading posture, and prolonged eye use in a dark environment could result in myopia among younger generations, especially in East Asia, where has the highest rate of blindness due to refraction disorders in terms of age-standardized DALY rates [24]. The overuse of electronic products and the lacks of outdoor activity time also contribute to the high incidence and low age of myopia [6]. We find that access to electricity could also affect the health burden of refraction disorders. The results of this studies may help to make better health policies that promote the SDGs.

It has been found that females were more vulnerable to health burden and vision loss due to refraction disorders than males, and the gender inequality would be influenced by age and socioeconomic levels. No significant difference of gender inequality is found in high SDI region in terms of age-standardized prevalence rates, which set an example for other countries. Women also suffer more eye diseases which occur late in life, such as presbyopia, the age-related loss of accommodation [25]. The gender inequality, especially among old people, may because that women have less access to eye care services than men, and the life expectancy of women is longer. Vision loss will not only reduce educational chances and the quality of life, but also cause productivity loss that might increase income gap between men and women [26]. In this way, eye care services should be emphasized more on old females. It should be noticed that the gender disparity has appeared among people in their thirties, and more studies are needed to analyze gender inequality in the next three decades in order to fulfill gender equality, because erasing the gender disparity is an important part towards SDGs.

Limitations of this study should be also noted. Since the research is subject to the methodological defects of the GBD study 2019 and World Bank Open Data 2019, further exploration of the methods and data are required to cover with the annual update of the databases. The absence of relevant data in some countries might lead to bias in the model estimates, and the number and quality of current data is not yet enough that may influence the accuracy. Second, different clinical procedures were used to measure visual acuity, which may increase measurement errors, and estimates before 2005 might be uncertain due to limited knowledge and data. Moreover, some people may suffer from multipile diseases rather than refraction disorders, which makes it difficult to determine the main cause of vision loss. Another limitation is that COVID-19 has changed the lifestyles of people around the world, which may causes the prediction of ARIMA model less precise.

In summary, this study finds that the global health of refraction disorders is improving, though an increasing and aging population keeps crude DALY rates stable. But this doesn’t mean fewer demands of refractive services. More quality refractive services should be given to females, elder population, and people in lower SDI regions. This study may raise public awareness of refraction disorders burden and is important for health policy making, which may help us to fulfill the SDGs in time.

## Data Availability

Data was acquired from the Global Health Data Exchange (http://ghdx.healthdata.org/gbd-results-tool), and Word Bank open data 2019 (http://data.worldbank.org/).

## Abbreviations

DALY: Disability adjusted life years
GBD: Global Burden of Disease
WHO: World Health Orgnization
SDGs: Sustainable Development Goals
YLD: Years lived with disability
YLL: Years of life lost
SDI: Socio-demographic Index
MSVI: Moderate and serve visual acuity
VA: Visual acuity
ARIMA: Auto-Regressive Integrated Moving Average
UI: Uncertainty intervals
